# Chaya Leaf Decreased Triglycerides and Improved Oxidative Stress in Subjects With Dyslipidemia

**DOI:** 10.3389/fnut.2021.666243

**Published:** 2021-07-23

**Authors:** Martha Guevara-Cruz, Isabel Medina-Vera, Trinidad Eugenia Cu-Cañetas, Yusef Cordero-Chan, Nimbe Torres, Armando R. Tovar, Claudia Márquez-Mota, José Moisés Talamantes-Gómez, Carlos Pérez-Monter, Roberto Lugo, Ana Ligia Gutiérrez-Solis, Azalia Avila-Nava

**Affiliations:** ^1^Departamento de Fisiología de la Nutrición, Instituto Nacional de Nutrición y Ciencias Médicas Salvador Zubirán, Ciudad de México, Mexico; ^2^Tecnologico de Monterrey, Escuela de Medicina y Ciencias de la Salud, Monterrey, Mexico; ^3^Departamento de Metodología, Instituto Nacional de Pediatría, Ciudad de México, Mexico; ^4^Escuela de Salud, Universidad Modelo, Mérida, Mexico; ^5^Departamento de Nutrición Animal y Bioquímica, Facultad de Medicina Veterinaria y Zootecnia, Universidad Nacional Autónoma de México (FMVZ-UNAM), Ciudad de México, Mexico; ^6^Departamento de Gastroenterología, Instituto Nacional de Nutrición y Ciencias Médicas Salvador Zubirán, Ciudad de México, Mexico; ^7^Hospital Regional de Alta Especialidad de la Península de Yucatán, Mérida, Mexico

**Keywords:** Chaya, dyslipidemia, antioxidant activity, oxidative stress, triglycerides

## Abstract

Chaya is an edible leaf popular in Mexico and Central America because of its high nutritional value. Studies in animal models have demonstrated the beneficial effects of Chaya, which include reduction of circulating lipids and increase in antioxidant activity. However, its hypolipidemic and antioxidant effects have not been demonstrated in humans. Thus, the aim of the present study was to evaluate the effect of Chaya on the lipid profile, lipid peroxidation, inflammation, and peripheral blood mononuclear cell gene expression in a population with dyslipidemia. We performed a single-arm trial in 30 participants with dyslipidemia who consumed 500 mL of Chaya beverage per day over a 6-week period. Interestingly, we observed a significant decrease in serum triglyceride concentration (*P* < 0.05) and an increase in plasma antioxidant activity and polyphenol concentration (*P* < 0.005) after 6 weeks of Chaya consumption. This was accompanied by a reduction in the oxidative stress marker MDA (*P* < 0.0001) and by an increase in the antioxidant enzyme *CAT* expression in peripheral blood mononuclear cells (*P* < 0.001). Altogether, our results demonstrate that consumption of Chaya has hypotriglyceridemic and antioxidant effects in subjects with dyslipidemia.

**Graphical Abstract:**
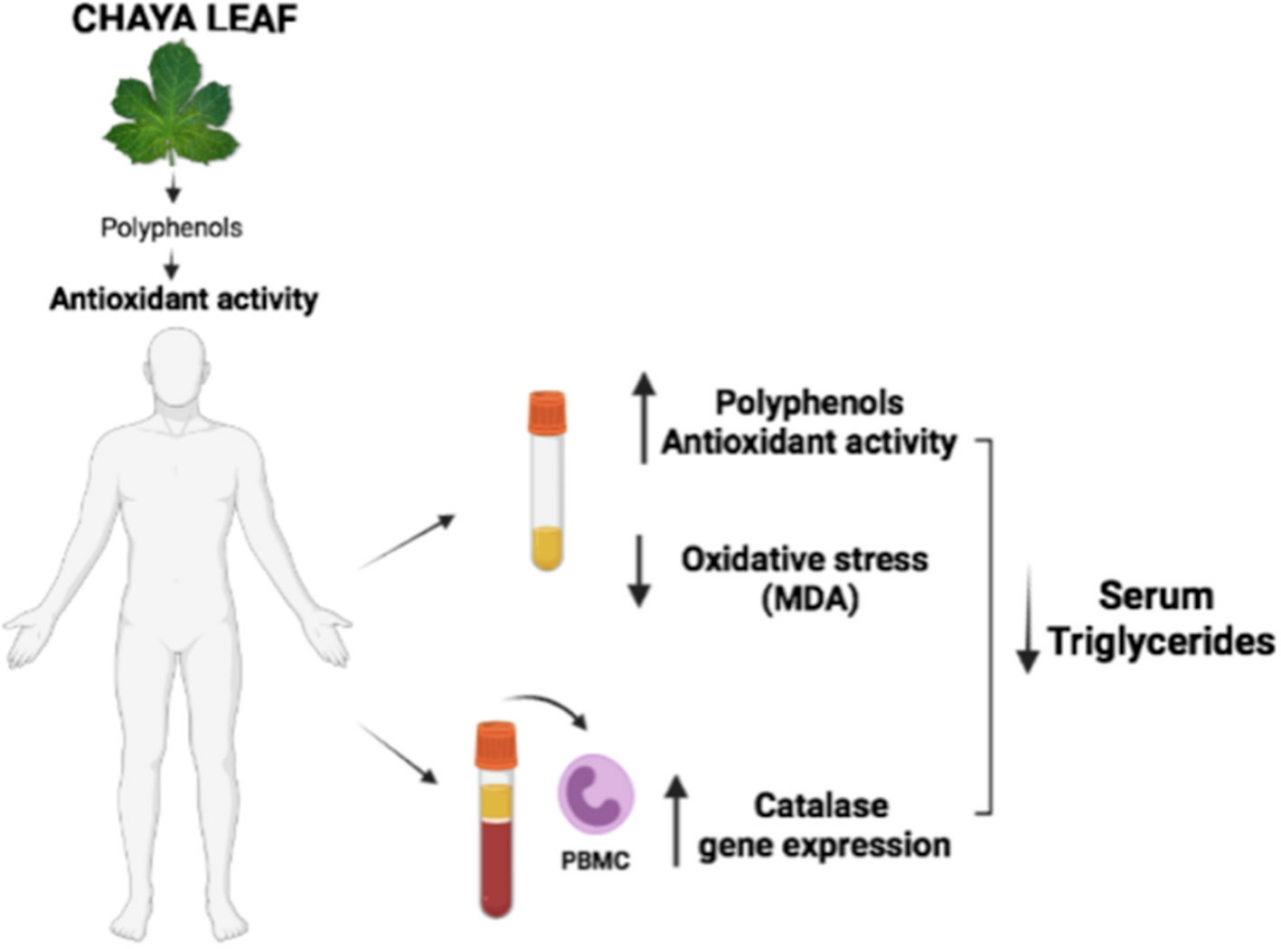
Effect of Chaya leaf on triglycerides, antioxidant activity and oxidative markers in subjects with dyslipidemia.

## Introduction

Dyslipidemia is characterized by abnormal lipid profiles that include increased levels of triglycerides (TG), total cholesterol (TC), low-density lipoprotein cholesterol (LDL-C), and/or decreased levels of high-density lipoprotein cholesterol (HDL-C). Dyslipidemia is one of the most important risk factors in formation of atherogenic plaques and development of cardiovascular diseases (CVD). Furthermore, hyperlipidemia is related to other complications, such as insulin resistance, endothelial dysfunction, hypertension and oxidative stress (OS) (1). In the context of OS, the increase in reactive oxygen species (ROS) promotes the oxidation of different biomolecules such as proteins, DNA, and lipids (lipoperoxidation). Malondialdehyde (MDA) is an end product of lipoperoxidation, which increases the level of pro-inflammatory cytokines such as interleukins and C-reactive protein (CRP) to establish a pro-atherogenic environment that results in endothelial dysfunction (2). These complications occurring in the context of dyslipidemia are due in part to a decrease in the expression and activity of antioxidant enzymes such as catalase (CAT) and superoxide dismutase (SOD), which enhances OS (3).

One potential strategy to improve lipid profiles is the consumption of foods with a high content of potential bioactive compounds (4). Chaya *(Cnidoscolus aconitifolius (Mill.) I.M. Johnst)* is an edible leaf popular in Mexico and Central America for its potential use as a vegetable and/or as a medicinal plant. It is widely used as traditional remedy for the treatment of obesity, diabetes, gastrointestinal disorders, and kidney stones (5). Chaya is also an important component of the regular diet of indigenous communities because of its nutritional value; it contains dietary fiber, protein, minerals, vitamins A and C, flavonoids, and polyphenols. Kaempferol and quercetin are the most abundant phenolic compounds identified in Chaya (6). Studies in animal models have demonstrated the effects of Chaya, which included reduction of glucose intolerance (7), anti-inflammatory effects, antioxidant activity (8), and hypolipidemic effects (9). These effects are highly great relevant to the current worldwide epidemic of dyslipidemia; however, there are no human studies demonstrating hypolipidemic or antioxidant effects of Chaya. Thus, the aim of the present study was to evaluate the effect of Chaya consumption on the lipid profile, lipid peroxidation, inflammation, and gene expression in subjects with dyslipidemia.

## Materials and Methods

### Plant Collection

Chaya *(Cnidoscolus aconitifolius (Mill.) I.M. Johnst)* was collected in Mérida, Yucatán from March to June 2019. The qualified botanist José L. Tapia-Muñoz identified plant and a voucher specimen (72343) was deposited at the herbarium of the Centro de Investigación Científica de Yucatán.

### Chemical Composition of the Beverage of Chaya and HPLC Analysis

The chemical composition of Chaya was analyzed with techniques recommended by the Association of Official Analytical Chemists (10). Antioxidant activity of Chaya was evaluated by oxygen radical absorbance capacity (ORAC) (11). The total polyphenol concentration was determined in Chaya using the Folin–Ciocalteu method (12). Additionally, we determined the phytochemical profile of a sample of Chaya with High-performance liquid chromatography (HPLC) system (Agilent 7 Technologies 1100) were done using a C18 column (Thermo Scientific ODS Hypersil) (250 × 4.6 mm, 5.0 μm). For HPLC analysis, a 2 g dry sample was obtained from beverage. Extraction was carried out using 2 g of dry sample with 30 mL of ethanol (85%) at 60 kHz in an ultrasonic extraction device, and repeated three times. The extract was stored at −4°C overnight. Then, 15 mL of methanol and 1.5 mL of hydrochloric acid were added and left to reflux for 2 h at 60°C. The solvent was removed on a rotary evaporator. The dried extract was dissolved in methanol; and approximately 1 mL of the extraction was filtered through a 0.45μm membrane and transferred into HPLC vials. The sample was eluted at a 1 mL/min flow rate with CH3OH: CH3CN: H2O (40:15:45) as a gradient mobile phase with 1% of acetic acid and detected by absorbance at 368 nm using a Waters 2998 photodiode array detector (13). The calibration curves of quercetin and kaempferol (Sigma, St. Louis, MO) were constructed using serial dilutions in ethanol of the standard solution (0.01, 0.05, 0.15, and 0.30 mg/mL). The sample was analyzed in triplicate.

### Subjects

Subjects with dyslipidemia characterized by TG >150 mg/dL, TC >200 mg/dL and/or LDL-C >120 and who were aged 20–60 were recruited through advertisements at the Hospital Regional de Alta Especialidad de la Península de Yucatán. Participants did not receive pharmacological treatment for the control of lipids. Exclusion criteria for these participants included TG ≥ 350 mg/dL, diabetes, a history of cardiovascular events, and weight loss of 3 kg within the last 3 months, cancer, acquired immunodeficiency syndrome, kidney or liver disease, smoking, substance abuse, and alcohol consumption. The study was approved by the Ethics Committee of the Hospital Regional de Alta Especialidad de la Península de Yucatán (No. 2018-030), Instituto Nacional de Pediatría (No. 2019/03), and registered at www.clinicaltrials.gov (NCT04110392), and conducted in accordance with the Declaration of Helsinki. Written informed consent was obtained from all participants.

### Sample Size

The required sample size was estimated to be 21 participants based on an expected effect of a 10% difference with 90% power and an α error of 0.05 for detecting effects on serum lipids resulting from the consumption of Chaya. The effect size was estimated based on studies of lipid-lowering supplements in patients with dyslipidemia (14), and a loss of 30% was expected during the study follow up, resulting in selecting 30 participants for enrollment in the study.

### Study Design

**Figure 1:**
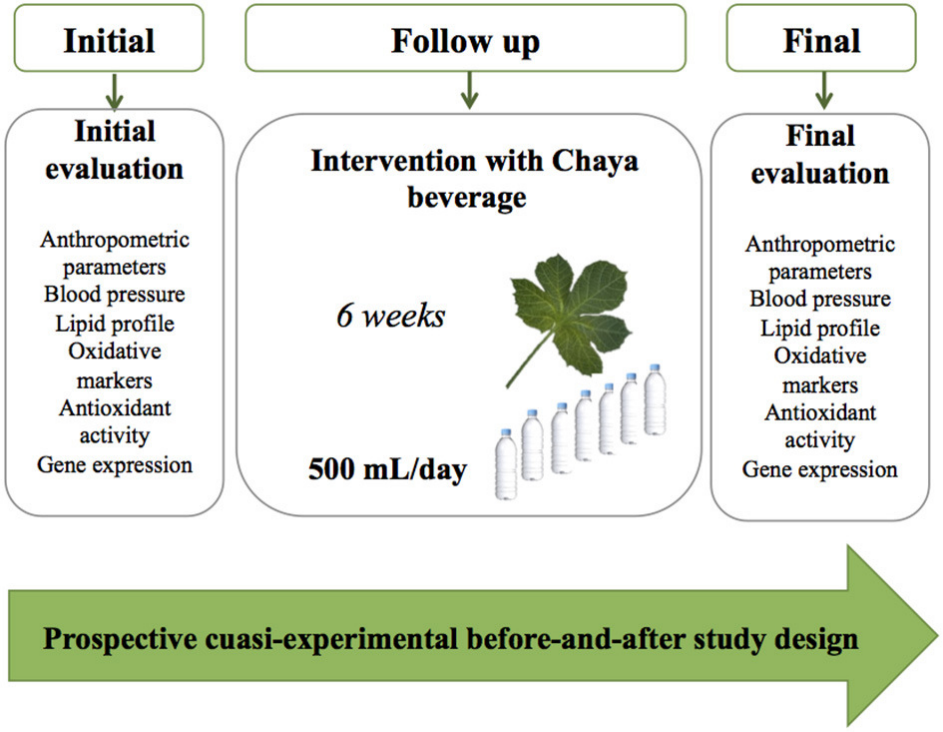
Study design.

The study had a before-and-after design to evaluate the effect of 6 weeks of Chaya beverage consumption in patients with dyslipidemia (Figure 1). At the first visit, a screening evaluation was conducted to select the participants based on the inclusion criteria. Eligible subjects received seven bottles of Chaya beverage to last 1 week. The Chaya beverage was prepared per week as follows: 40 g of Chaya leaves, which were soaked, disinfected, and mixed with 1 L of purified water. Finally, 500 mL of the beverage was placed in bottles and stored at −4°C until consumption during 1 week. At each visit (weekly), seven bottles and a daily consumption form were given to participants, and the participants were instructed to drink one bottle all at once per day for 1 week and record it on the form. At the end of each visit, participants were asked to return the empty bottles and the daily consumption form. The participants were advised to maintain their normal lifestyle, activity level and diet, and were instructed to restrict the consumption of foods containing Chaya. At the baseline and end of the study, body weight, height, and waist circumference were determined by the Lohman method (15). The waist-to-height ratio (WHtR) was calculated by dividing the waist circumference (cm) by the height (m). Blood pressure was measured with a digital automatic blood pressure monitor (HEM-6122 LA, Omron Healthcare, Inc., Sunrise, FL, USA). Blood samples were collected after 8–10 h of fasting at the baseline and at the end of the study. Briefly, blood samples were centrifuged at 500x*g* for 5 min, and serum and plasma were maintained at −70°C until analysis. Serum TC, TG, LDL-C, HDL-C, and glucose were analyzed with a COBAS C11 (Roche Applied Science).

### Oxidative and Inflammation Markers

Serum MDA concentration was measured as a marker of lipid peroxidation by a spectrophotometric method as previously reported (16), and CRP was analyzed with the COBAS C11 (Roche Applied Science).

### Determination of Antioxidant Activity and Total Polyphenols

Antioxidant activity of plasma was evaluated by ORAC (11), and the total polyphenol concentration was determined in plasma using the Folin–Ciocalteu method (12).

### Peripheral Blood Mononuclear Cell Gene Expression by Quantitative Real-Time PCR

Total RNA was extracted from peripheral blood mononuclear cells (PBMC) using TRIzol according to the manufacturer's instructions. The mRNA abundance was measured by real-time quantitative PCR using SYBR®Premix, using actin as a reference for normalization. The primers are listed in Supplementary Table 1. The relative expression was calculated using the ΔΔCt method (17).

### Statistical Analysis

Continuous variables were evaluated using the Kolmogorov-Smirnov test to analyze the type of distribution. Wilcoxon tests were performed for paired samples to compare the medians of the lipid profile, glucose, CRP, antioxidant activity, oxidative markers, and body weight at baseline and after the intervention. For gene expression, logarithmic transformation was performed before analysis with a dependent-samples *t-*test. A value of *P* < 0.05 was considered significant. Correlations between two variables were evaluated by Spearman's test. Data are presented as the mean ± standard deviation (SD) or standard error of the mean (SEM); or medians and 95% confidence intervals (95% CI). Data were analyzed using SPSS version 25 software for Macintosh (IBM Corp., Armonk, NY, USA).

## Results

### Analysis of Chemical Composition and Identification of Bioactive Compounds in Chaya Beverage

**Figure 2:**
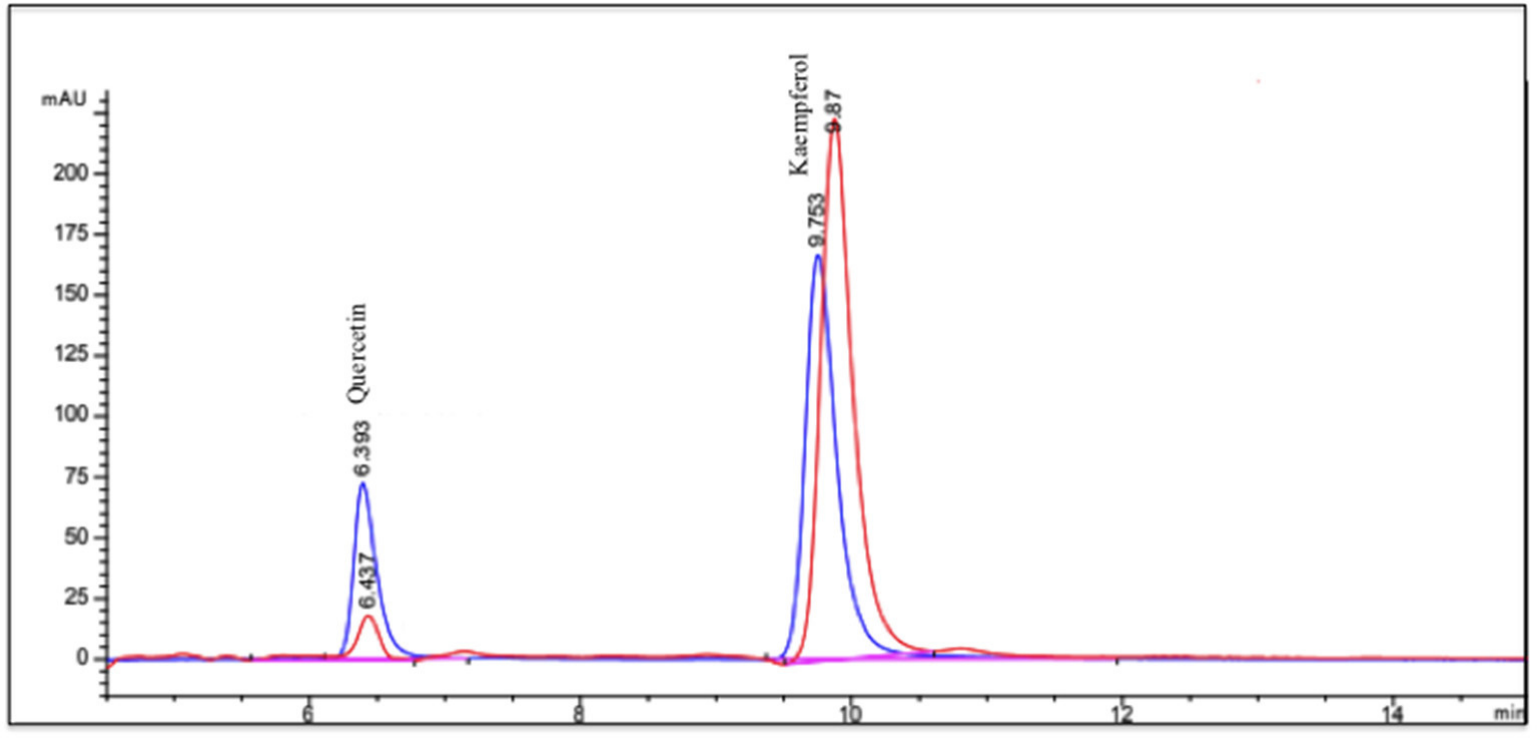
HPLC profile of Chaya. Principal compounds in Chaya are quercetin (6.43 min) and kaempferol (9.87 min) (red line).

The Chaya beverage mainly contained protein, fiber, and minerals (Table 1). Its antioxidant activity was 807 ± 10.53 Trolox equivalents (μmoles/L), and the concentration of polyphenols was 1,359 ± 47.2 mg of gallic acid equivalents (GAE)/L. The HPLC profile obtained showed a pattern with two peaks that represented the quercetin and kaempferol present in Chaya (Figure 2). The retention time of quercetin was determined at 6.39 min and kaempferol at 9.75 min. The concentration of quercetin in the Chaya beverage was 0.21 ± 0.008 mg/mL and kaempferol was 1.72 ± 0.015 mg/mL.

**Table 1:** Chemical composition of the beverage Chaya. Data are expressed as a portion of daily intake (20 g/500 mL). Values represent the mean ± SEM.

	**Content (g/500 mL)**
Protein	1.02 ± 0.02
Carbohydrates	0.59 ± 0.05
Fiber	0.58 ± 0.03
Minerals	0.38 ± 0.01
Lipids	0.18 ± 0.02

### Anthropometric Parameters, Blood Pressure, and Glucose Concentration in Subjects With Dyslipidemia

A total of 112 participants were screened, and 30 subjects were enrolled. The study population had a mean age of 42.5 ± 10.8 years, and 46.7% (*n* = 14) were male. At baseline, subjects had a body mass index (BMI) of 29.2 ± 4.41 kg/m^2^, and blood glucose of 89.1 (95% CI, 74.2–127) mg/dL. was 115 (95% CI, 98–180) and 74 (95% CI, 59–180) mmHg, respectively. After 6 weeks of the dietary strategy, the subjects with dyslipidemia who consumed the Chaya beverage exhibited a decrease in systolic BP (−3.5%) (*P* < 0.05) (Table 2). However, diastolic BP, glucose concentration, body weight, and BMI did not show differences after intervention. At the end of the study, a percentage of 94 ± 5.1 of adherence to the intervention was reported. The consumption of Chaya did not have adverse effects in the participants.

**Table 2:** Anthropometric characteristics, blood pressure, and biochemical parameters at baseline and after intervention with Chaya.

**Characteristic**	**Baseline**	**Week 6**	***P*-value**
Weight (kg)	72.2 (50.4–115)	72.3 (50.1–117)	0.050
BMI (kg/m^2^)	29.2 ± 4.41	29.1 ± 4.41	0.160
Circumference of waist (cm)	93 (74.1–120)	93 (72.8–118)	0.292
WHtR	0.58 (0.46–0.69)	0.59 (0.48–0.68)	0.791
Systolic BP (mmHg)	115 (98–180)	112 (93.4–166)	0.032
Diastolic BP (mmHg)	74 (59–180)	73 (61.7–106)	0.992
Glucose (mg/dL)	89.1 (74.2–127)	91.9 (80.1–107)	0.052
Triglycerides (mg/dL)	208 (114–301)	166 (90–328)	0.049
TC (mg/dL)	188 (143–264)	186 (149–260)	0.133
HDL-C (mg/dL)	39.7 (27.6–70.3)	41.5 (28.9–58.7)	0.425
LDL-C (mg/dL)	121 (72.7–200)	120 (95.1–185)	0.632

*Values represent the mean ± SD or median (95% confidence interval). Body mass index, (BMI); Waist-to-height ratio (WHtR); Blood pressure (BP); Total cholesterol (TC); Highdensity lipoprotein cholesterol (HDL-C); Low-density lipoprotein cholesterol (LDL-C). Statistical analysis was determined by the Wilcoxon test or dependent-samples t-test*.

### Lipid Profiles in Subjects Before and After Chaya Beverage Intervention

The subjects with dyslipidemia included in this study showed lipid alterations in TG (208 mg/dL; 95% CI, 114–301) and LDL-C (121 mg/dL; 95% CI, 72.7–200). After consuming Chaya for 6 weeks, there was a significant reduction of 20.6 % in the concentration of TG (*P* < 0.05) (Table 2). In contrast, circulating levels of TC, LDL-C, and HDL-C did not show differences after Chaya consumption.

### Chaya Consumption Enhanced Antioxidant Activity in Subjects With Dyslipidemia

**Figure 3:**
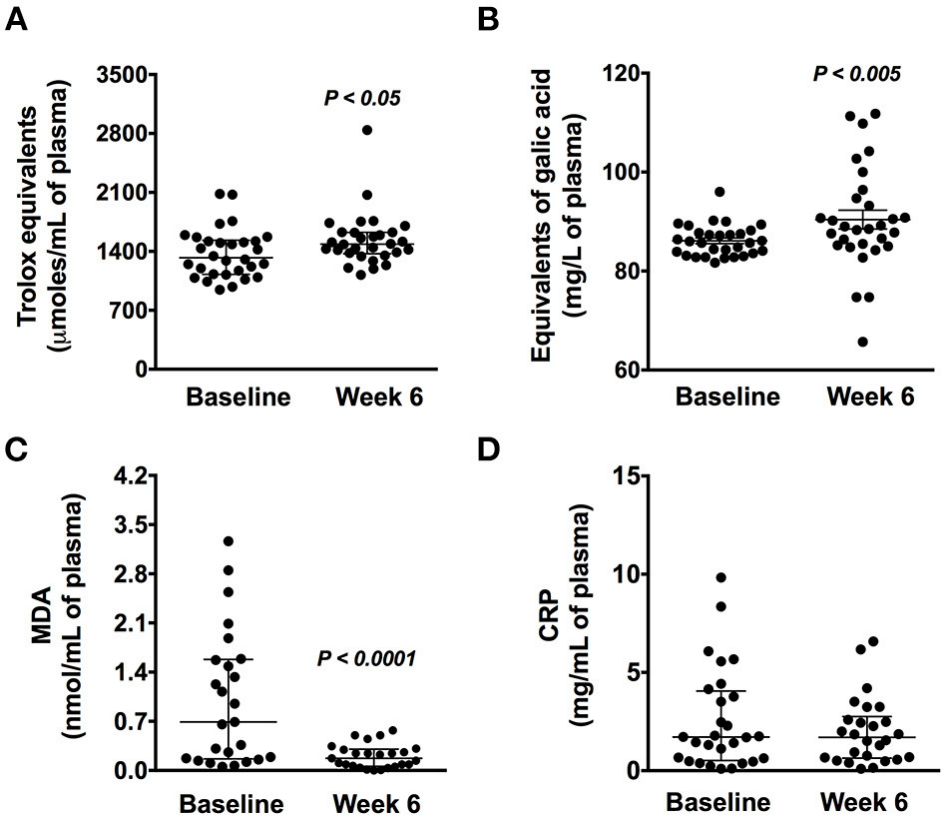
Oxidative and inflammation markers in subjects with dyslipidemia at baseline and before Chaya intervention. **(A)** Antioxidant activity. **(B)** Plasma polyphenol concentration. **(C)** Plasma malondialdehyde. **(D)** C-reactive protein concentration. Data are expressed as the median (95% confidence interval) (*n* = 30). Statistical analysis was performed using Wilcoxon tests.

**Figure 4:**
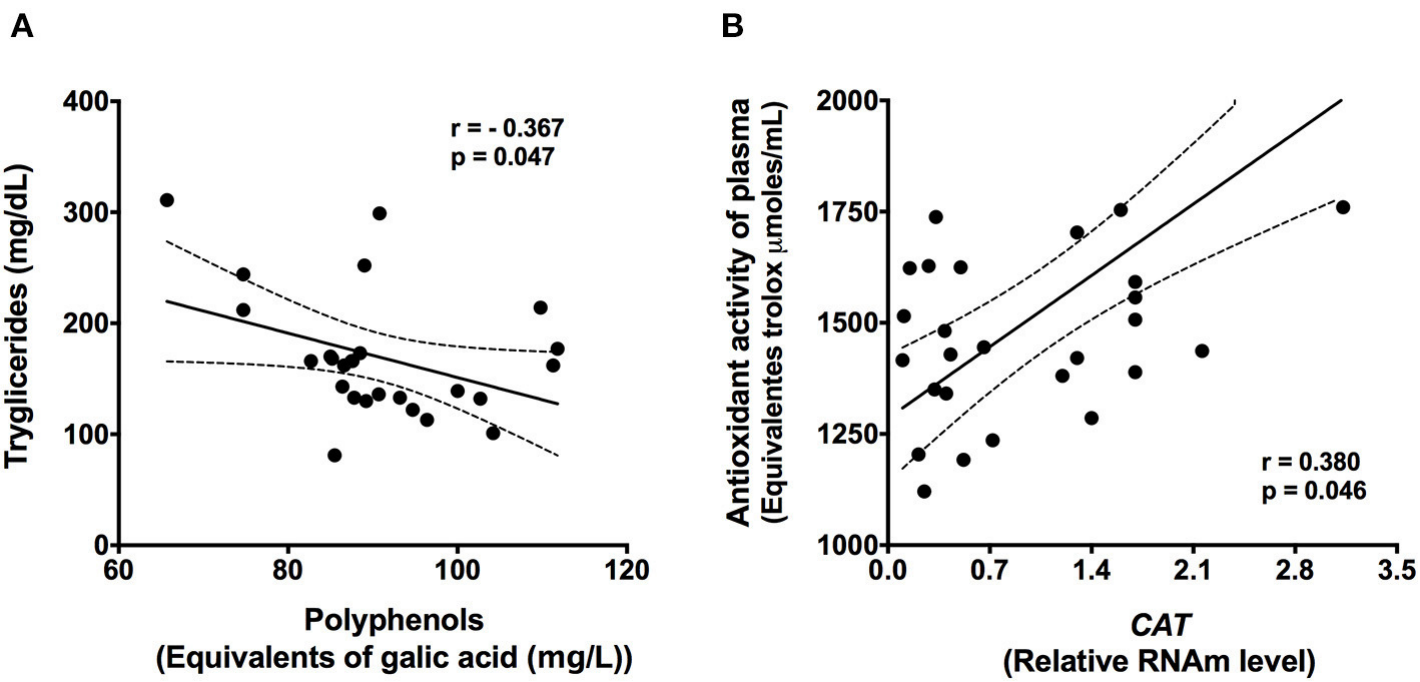
Correlations between serum triglycerides and plasma polyphenols **(A)** and between plasma antioxidant activity and CAT gene expression **(B)** (*n* = 30). Statistical analysis was performed by Spearman's test (95% confidence interval).

Interestingly, the antioxidant activity increased in plasma from 1324 Trolox equivalents (μmoles/mL) (95% CI, 963–2078) to 1485 (95% CI, 1160–2416) (*P* < 0.05) (Figure 3A). This was accompanied by a significant increase of 4% in the plasma polyphenol concentration (*P* < 0.005) (Figure 3B). Furthermore, subjects with dyslipidemia showed an inverse correlation between plasma polyphenols and serum TG (*r* = −0.367, *P* = 0.047; Figure 4A).

### Chaya Intake Decreased in Oxidative Marker MDA

Notably, the 6-week Chaya intervention reduced the MDA concentration by 80.4% (*P* < 0.0001) (Figure 3C) with no changes in the inflammatory marker CRP (Figure 3D). There is evidence of a relationship between OS and inflammation and the development of CVD and mortality (18, 19).

### Differential Expression in Peripheral Blood Mononuclear Cells of Antioxidant and Lipid Metabolism Genes Resulting From Chaya Consumption in Subjects With Dyslipidemia

**Figure 5:**
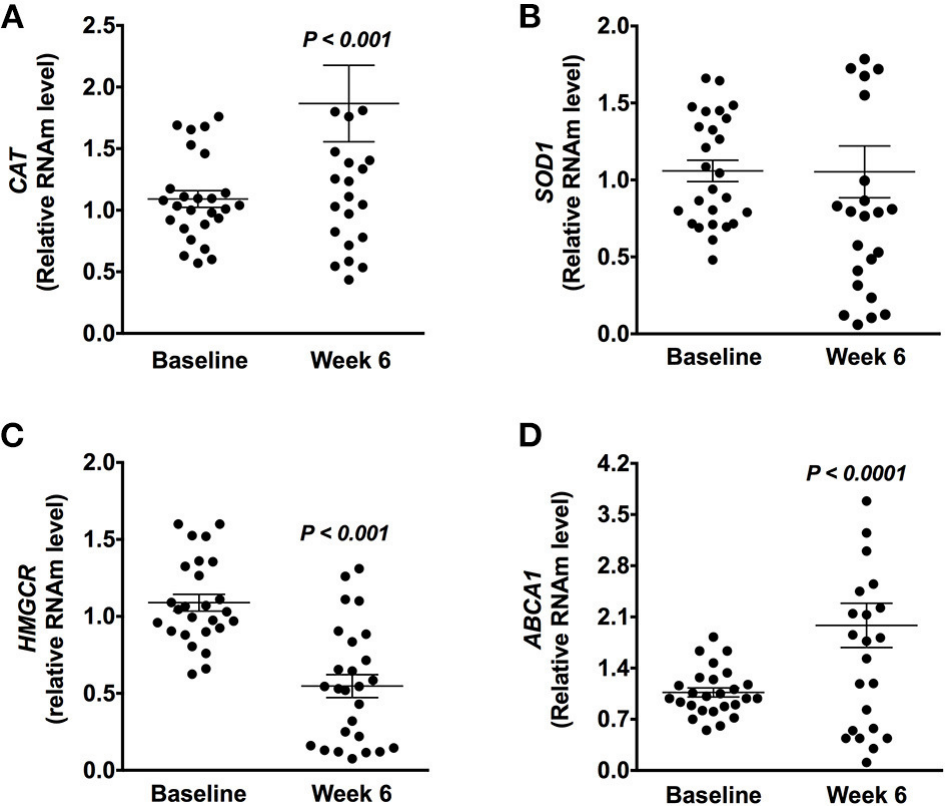
Differential expression in peripheral blood mononuclear cell of genes involved in antioxidant activity and cholesterol flux modulated by Chaya intake. **(A)** Relative gene expression of *CAT* (catalase). **(B)** Relative gene expression of *SOD* (Superoxide dismutase 1). **(C)** Relative gene expression of *HMGCR* (hydroxy-methyl-glutaryl coenzyme A reductase). **(D)** Relative gene expression of *ABCA1* (ATP-binding cassette transporter A1). Data are expressed as the mean ± SD. Statistical analysis was performed using a paired *t*-test after logarithmic transformation.

As expected, the antioxidant enzyme *CAT* showed a significant increase after Chaya intake (*P* < 0.001) without changes in the expression of *SOD1* (Figures 5A,B) in PBMC. In fact, there was a positive correlation between the gene expression of *CAT* and plasma antioxidant activity (*r* = 0.380, *P* = 0.046; Figure 4B). In addition, the expression of lipid-related genes was evaluated. After the consumption of Chaya, there was a significant decrease in the gene expression of *HMGCR* (hydroxy-methyl-glutaryl coenzyme A reductase) in PBMC, which is involved in hepatic cholesterol synthesis (Figure 5C), and this decrease was accompanied by an increase in the gene expression of *ABCA1* (ATP-binding cassette transporter A1) (Figure 5D), which mediates the efflux of cholesterol to regulate lipid levels.

## Discussion

Our results demonstrated that a nutritional strategy that includes the consumption of a Chaya beverage for 6 weeks reduced plasma triglyceride concentration and lipoperoxidation, increased plasma antioxidant activity, and modulated the expression of antioxidant and lipid metabolism genes in PBMC in subjects with dyslipidemia.

Residents in middle-income countries experience health disparities and less healthy eating patterns; thus, it is very common for inhabitants to use traditional foods or herbs to control different types of diseases. Chaya leaves are used in Mayan traditional medicine to treat kidney stones, diabetes, and high BP; infusions or teas make its administration from Chaya leaves. However, communities use native food components to control several diseases mainly based on empirical knowledge of their effects on health (20). The relevance of our work resides in the evaluation of a dietary strategy that included the use of Chaya, an inexpensive and native regional food, to decrease lipid alterations. A few studies *in vitro* and in animal models have demonstrated antioxidant, anti-inflammatory, hypoglycemic, and hypolipidemic effects of Chaya (8, 21). In fact, a study in rats showed that doses of 0.5–1.5 mg/kg of Chaya extracts administered intragastrically decreased the concentrations of TG and TC (9). Interestingly, in the present study, we observed an inverse correlation between plasma polyphenols and serum TG, supporting the hypothesis that the hypolipidemic effects of Chaya consumption may be attributed to its effect on polyphenols. This lipid-lowering effect promoted by polyphenols could be attributed to the activation of AMP-activated protein kinase (AMPK) and inactivation of acetyl-CoA carboxylase, which in turn promotes reduced fatty acid synthesis and increased fatty acid oxidation (22). *In vitro* and *in vivo* studies have shown that polyphenols, such as quercetin and kaempferol, reduce plasma TG by regulating fatty acid mobilization and triglyceride synthesis (23–25). In addition, AMPK activation also involves an increase in energy expenditure, reduction in lipogenesis, and reduction in fat mass and OS through activation of its energetic sensors (26–28). However, the effects of polyphenols in our study were not associated with changes in body weight, which could be attributed to the dose used or to the time of intervention. The lack of effect on BMI has been previously observed with an intervention with 300 mL of blackberry juice drunk daily for 8 weeks (29). Conversely, our results suggest that the hypolipidemic effects of Chaya may also be related to the antioxidant effects of polyphenols. Studies have shown that persistent ROS generation is involved in lipid dysregulation caused by decreased fatty acid uptake, lipogenesis, and β-oxidation of fatty acids (30, 31). In fact, one study showed a statistically significant correlation between the oxidative stress index and serum cholesterol (*p* < 0.001; *r* = 0.596) and triglycerides (*p* < 0.001; *r* = 0.476) (32). Thus, the antioxidant activity of polyphenols reduces ROS production and also the increase of endogenous antioxidants ameliorates hyperlipidemia and the alterations of many cell or tissue types caused by OS in hyperlipidemia (33).

Our work is the first study conducted in humans to show the clinical hypotriglyceridemic and antioxidant effect of Chaya consumption. Notably, consumption of the Chaya beverage (40 g/L) showed antioxidant activity. These results are similar to the antioxidant effect resulting from the consumption of most antioxidant-rich foods, which are reference sources of polyphenols and widely known for their health-promoting properties (34–38). For example, one study demonstrated that intervention with Mate tea (1L/day) for 90 days promoted a significant increase in serum antioxidant activity compared to baseline levels in the population with dyslipidemia (*P* < 0.05) (35). Another study demonstrated a significant increase in total antioxidant capacity in subjects with hypercholesterolemia by daily consumption of red wine (125 mL for women or 250 mL for men) for 1 month (36). Another popular beverage that showed a similar antioxidant effect is coffee; its consumption (600 mL/day) for 8 weeks increased plasma antioxidant activity in subjects with hypercholesterolemia (37). In addition, orange juice consumption (750 mL/day) for 8 weeks increased antioxidant activity and decreased TC in overweight subjects (38). This is important as it has been shown that not all interventions involving antioxidant foods in subjects with dyslipidemia generated an effect on circulating antioxidant activity (39). This types of foods have received considerable attention because of their potential antioxidant activity by hydroxyl groups and double bonds in chemical structure of polyphenols (40). In addition, the consumption of different foods with high concentrations of polyphenols, such as beverages made from fruits and vegetables (41), blackberry juice (29) and red grapes (42), showed a significant reduction in the lipid profile of subjects with dyslipidemia. Despite the beneficial effects of these foods on dyslipidemia, most of them are not always accessible to different communities in accordance with their annual availability or to the lifestyle and traditions of the population of those communities. Therefore, based on our results, Chaya represent a low-cost nutritional strategy against dyslipidemias in subjects with access to this plant such as families in the state of Yucatan and also throughout Central America (43).

Interestingly, we observed that consumption of Chaya not only increased antioxidant activity but also decreased the levels of MDA in subjects with dyslipidemia, suggesting a protective effect against OS. Other foods that have been extensively studied for their antioxidant activity and reduction of the oxidative damage in lipids are orange juice (−55%) and coffee (−10.3%) (37, 38). The antioxidant activity of polyphenols can suppress the oxidative modification of plasma lipids and lipoproteins, which are major targets of many antiatherogenic agents and the preferable strategy to prevent the development of CVD. Furthermore, polyphenols, such as quercetin and kaempferol, which are also present in Chaya, can act by activating the transcription factor Nrf2, which travels to the nucleus to bind to the antioxidant response element sequence and induce the expression of genes of the endogenous antioxidant system such as *CAT* and *SOD* (44). Our results demonstrated that Chaya consumption induced an increase in *CAT* gene expression in PBMC, which is in agreement with previous evidence that dietary intervention with food rich in antioxidants can modulate antioxidant enzymes in subjects with dyslipidemia; for example, the consumption of the traditional Chinese herb *Graptopetalum paraguayense* significantly increased the enzymatic activity of glutathione peroxidase and CAT (45), and aqueous wolfberry fruit intake enhanced the activity of CAT (46). These results suggest that polyphenols present in Chaya contribute to maintaining the redox homeostasis of cells through the activation of antioxidant enzymes. Although an increase of polyphenols in plasma induced the antioxidant machinery, it was not enough to generate a decrease in inflammatory marker CRP, which could be attributed to the doses of the bioactive compound in Chaya. The anti-inflammatory effect is directly dependent of the doses of polyphenols. In fact, a systematic review and meta-analysis showed a tendency toward lower serum concentrations of inflammatory biomarkers only at dose of >200 mg/day of polyphenols from grapes (31) and only 90 mg in plasma (47).

PBMC gene expression could be used as a tool to assess potential markers of the mechanism of action of the intervention. Indeed, one study demonstrated that statins can regulate gene expression in PBMCs from patients with dyslipidemia before detectable changes in lipid profile occur (48). The intervention with Chaya significantly regulated *HMGCR* and *ABCA1* gene expression in PBMC, despite no differences observed in serum total cholesterol in our participants, such as was found for TG. In this sense, recent studies have suggested a function for ABCA1, especially macrophage ABCA1, in lipid metabolism (49). Evidence showed that gene expression of *ABCA1* is induced during differentiation of monocytes into macrophages and up-regulated by cholesterol influx. The proposed molecular mechanism is the induction of *ABCA1* gene expression by peroxisome proliferator-activated receptors -α and -γ in human macrophages and macrophage-derived foam cells, which promotes increased cholesterol flow from macrophages to the liver (50). Thus, this process could be control susceptibility to macrophage recruitment into different tissues and development of atherosclerosis (51).

Our results on the antioxidant and hypotriglyceridemic effects of Chaya consumption are very interesting. However, this study has some limitations, such as the study design and the small sample size. This type of study did not include a control group and age of participants was highly variable, but they are useful as a first approximation to evaluate the feasibility and acceptability of interventions. In addition, some factors that may affect the outcome measurements, such as dietary caloric restriction, were not examined in the present study. However, the intention of the study was to evaluate the effect of this intervention without modifying the lifestyle of the participants, which contributes to know the effect of Chaya and not of the other modifications that could have been suggested to the population. Thus it remains to be explored whether the findings of this study can be applied to other groups.

## Conclusion

Altogether, our results demonstrate that consumption of Chaya has hypotriglyceridemic and antioxidant effects in subjects with dyslipidemia.

## Data Availability Statement

The datasets generated for this study are available on request to the corresponding author.

## Ethics Statement

This study involving human participants was reviewed and approved by the Ethics Committee of the Hospital Regional de Alta Especialidad de la Península de Yucatán (No. 2018-030) and Instituto Nacional de Pediatría (No. 2019/03). The participants provided their written informed consent to participate in this study.

## Author Contributions

MG-C, IM-V, and AA-N designed the study. TC-C, YC-C, CM-M, and JT-G performed the experiments. CP-M, RL, and AG-S analyzed the data. AA-N, IM-V, NT, and AT wrote the paper. All authors contributed to the manuscript.

## Conflict of Interest

The authors declare that the research was conducted in the absence of any commercial or financial relationships that could be construed as a potential conflict of interest.

## Publisher's Note

All claims expressed in this article are solely those of the authors and do not necessarily represent those of their affiliated organizations, or those of the publisher, the editors and the reviewers. Any product that may be evaluated in this article, or claim that may be made by its manufacturer, is not guaranteed or endorsed by the publisher.

## Abbreviations

ABCA1: ATP-binding cassette transporter A1 gene
AMPK: AMP-activated protein kinase
BMI: body mass index
BP: blood pressure
CAT: catalase
CRP: C-reactive protein
CVD: cardiovascular diseases
HDL-C: high-density lipoprotein cholesterol
HMGCR: hydroxy-methyl-glutaryl coenzyme A reductase
HPLC: High-performance liquid chromatography
LDL-C: low-density lipoprotein cholesterol
MDA: Malondialdehyde
ORAC: oxygen radical absorbance capacity
OS: oxidative stress
PBMC: peripheral blood mononuclear cells
ROS: reactive oxygen species
SD: standard deviation
SEM: standard error of the mean
SOD: superoxide dismutase
TC: Total cholesterol
TG: triglycerides
WHtR: waist-to-height ratio
95% CI: 95% confidence intervals.
