# Supplementary material for: Chaya Leaf Decreased Triglycerides and Improved Oxidative Stress in Subjects With Dyslipidemia
Source: Front Nutr. 2021 Jul 23;8:666243. doi: 10.3389/fnut.2021.666243 (PMC8343181; doi:10.3389/fnut.2021.666243)
Supplement: Supplementary file 1 [file Table_1.docx]

**Supplementary material**

Table S1. Primers used in real-time PCR

| **Gen** | **Primer** | **Sequence** |
| --- | --- | --- |
| *CAT* | Forward  Reverse | TGTGCATGCAGGACAATCA  CCAGAATATTGGATGCTGTGC |
| *SOD1* | Forward  Reverse | TCCATGTTCATGAGTTTGGAGAT  CCCACCGTGTTTTCTGGATA |
| *HMGCR* | Forward  Reverse | AGAGGCTGCAGAGCAATAGG  CATCCCATCTGCAAGGACTC |
| *ABCA1* | Forward  Reverse | CACCCTTTGGCAAGTACCC  CTCAGGAGCATCATTGCTG |
| *ACTIN* | Forward  Reverse | AGAGCTACGAGCTGCCTGAC  CGTGGATGCCACAGGACT |
